# Dissemination of the 2022 ASMBS and IFSO Guidelines for Bariatric Surgery: What Has Reached Primary Care Providers?

**DOI:** 10.1007/s11695-024-07449-1

**Published:** 2024-08-15

**Authors:** John Hulse, Richard Slay, Mary Kate Bryant, T. Karl Byrne, Rana Pullatt

**Affiliations:** Medical University of South Carolina, Charleston, SC USA

**Keywords:** 2022 ASMBS, IFSO guidelines, Bariatric surgery

## Abstract

**Background:**

Only 1% of Americans eligible for metabolic and bariatric surgery (MBS) receive MBS. Prior studies have analyzed primary care provider (PCP) referral patterns and perceptions of MBS as a potential barrier to increasing MBS. However, less data exists regarding PCP knowledge of MBS indications and outcomes. Following the 2022 update to the indications for MBS by the ASMBS and IFSO, the number of eligible patients is only expected to increase. We evaluated PCP knowledge regarding the existence of the 2022 ASMBS and IFSO updated guidelines, MBS indications, and MBS outcomes.

**Methods:**

An 11-question survey was emailed to primary care residents, advanced practice providers, and faculty at a single institution.

**Results:**

Of 151 surveys distributed, 39.7% responded (*n* = 60). 95% were unaware of the 2022 updated guidelines. On multiple choice questions, 16.3% correctly identified the average weight loss from MBS, and 46.8% correctly answered the diabetes remission rate following MBS. Trainee answers were not statistically significant from practicing PCPs. Fifteen respondents had referred a patient for MBS, but this subgroup did not perform significantly better on the assessment. A total of 72.3% of respondents reported inadequate MBS education during their training, and 85.1% were interested in additional education.

**Conclusions:**

We present the first assessment of PCP MBS knowledge since the release of the 2022 updated ASMBS and IFSO guidelines. This study indicates a gap in PCPs’ knowledge regarding the updated guidelines and represents an opportunity for collaboration with our primary care colleagues to provide further MBS education.

**Supplementary Information:**

The online version contains supplementary material available at 10.1007/s11695-024-07449-1.

## Introduction

**Fig. 1:**
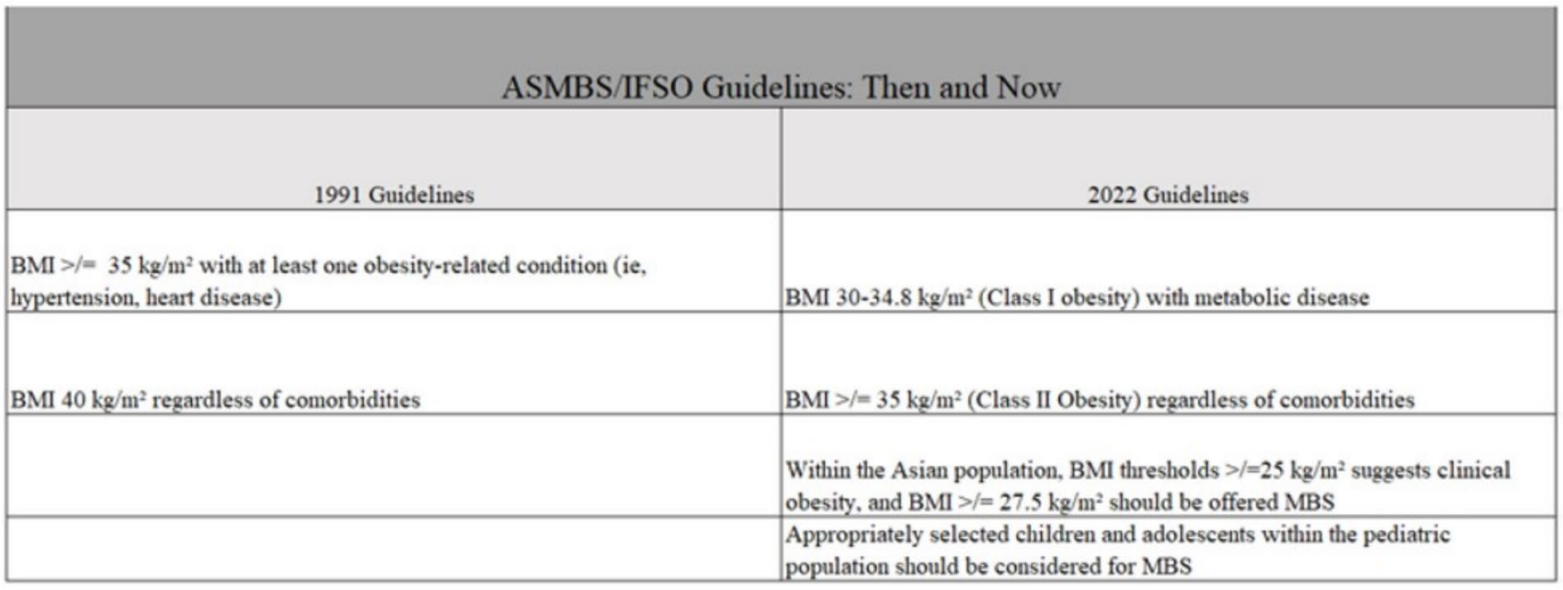
Comparison of 1991 and 2022 American Society of Metabolic and Bariatric Surgery (ASMBS) and International Federation for the Surgery of Obesity and Metabolic Disorders (IFSO) guidelines

Even with improvements in the safety and efficacy of bariatric surgery, less than 1% of United States adults meeting the BMI criteria opt for bariatric surgery [1]. A disconnection clearly exists between patients eligible for metabolic and bariatric surgery (MBS) and those who eventually undergo surgery. The number of eligible patients is expected to rise following the 2022 American Society of Metabolic and Bariatric Surgery (ASMBS) and International Federation for the Surgery of Obesity and Metabolic Disorders (IFSO) updated guidelines. In contrast to the prior ASMBS guidelines outlined by the NIH consensus panel in 1991, the new guidelines released in October 2022 lower the BMI thresholds at which MBS referral is suggested in the general population, create separate guidelines for the Asian population, and include the pediatric population [2] (Fig. 1).

Retrospective studies have yielded two sources for the discrepancy between patients eligible for MBS and those who undergo MBS: patient factors and provider factors. In a qualitative study by Murtha et al., multiple patient factors that were identified include fear of surgery and its outcomes, fear of lifestyle change, perception that an individual’s weight had not reached its tipping point, concerns about adhering to dietary changes, and lack of social support [3]. Despite advancements in surgical technique and low rates of mortality of less than half of a percentage point [4], patients remain concerned regarding the safety of MBS. Primary care providers (PCP) may be the first medical professional point of contact for patients exploring the safety and efficacy of MBS. However, patient factors are not well-studied outside of single-institution qualitative research.

Provider-centered factors relating to the paucity of those patients eligible for MBS who undergo surgery are better studied. Survey studies have been performed focusing on barriers to PCP referrals. A 2019 survey study performed by Lopez et al. noted the top three reasons indicated by all providers for being uncomfortable broaching MBS discussions included questions regarding insurance coverage, concerns regarding patient eligibility for surgery, and insufficient knowledge to educate patients on their options [5]. A 2020 study performed by Conaty et al. found that concerns regarding the possible medical and surgical complications that may result from MBS have limited providers’ willingness to discuss the potential for bariatric surgery with their patients [6]. Tork et al. highlight a final important barrier: providers who care for patients eligible for MBS have concerns about managing MBS surgical patients postoperatively, i.e., tracking and counseling weight loss following surgery, identifying complications, and managing nutritional status [7]. This is further complicated by the estimation that up to 10% of patients who have undergone MBS did not attend a 2-year follow-up with a bariatric center [8]. Loss of follow-up in such a life-altering procedure can lead to failed identification and treatment of postoperative complications along with an increased burden placed on PCPs.

As mentioned above, with the broadened MBS referral guidelines in the 2022 ASMBS and IFSO guidelines, the number of eligible patients for MBS is expected to increase significantly. Few surveys include an assessment of the provider knowledge base, and fewer studies have evaluated knowledge and perceptions at a trainee level. In our survey-based study, we report the first study to evaluate PCP knowledge regarding the existence of the 2022 ASMBS and IFSO updated guidelines, MBS indications, and MBS outcomes. We hypothesized that knowledge of the updated guidelines would be low but would be higher amongst independent practitioners and those who had previously referred a patient for bariatric surgery.

## Materials and Methods

An eleven-question survey was created using REDCap (see supplemental material). The survey included multiple choice questions, yes/no questions, and multiple response questions. The survey primarily assessed PCP knowledge of bariatric surgery outcomes and the 2022 ASMBS and IFSO updated guidelines. An additional question queried prior referral practice of patients with obesity for MBS evaluation. The survey provided the correct answers to each question with an explanation following each question. The respondent was required to answer each question to advance through the survey, but if the respondent exited out of the survey prior to completing all questions, the answers they had submitted were saved and recorded.

This survey was distributed via email to the Medical University of South Carolina’s (MUSC) internal medicine and family medicine departments, including a total of 151 people. The responses were collected over the period from May 2023 to October 2023. This included sixteen internal medicine faculty, three internal medicine advanced practice providers (APPs), 103 internal medicine residents, and 29 family medicine residents. Survey results were anonymous, but the survey did inquire about the duration the respondent had been practicing and distinguished residents from faculty and APPs.

MUSC is a nationally accredited metabolic and bariatric surgery center of excellence reviewed by the MBS Accreditation and Quality Improvement Project (MBSQIP), a joint program with the American College of Surgeons (ACS) and ASMBS. This study was deemed exempt as a quality improvement project through the MUSC Institutional Review Board (IRB).

Quantitative survey data were analyzed using descriptive statistics to report the frequencies of specific answers. Subgroup analyses were performed to analyze answers for trainees versus independent practitioners and those who had referred a patient previously to bariatric surgery versus not. For all analyses, a *p*-value ≤ 0.05 was considered statistically significant. Missing data were excluded from calculations that were specific to that field. Analyses were performed using Stata 16 (College Station, Texas).

## Results

**Fig. 2:**
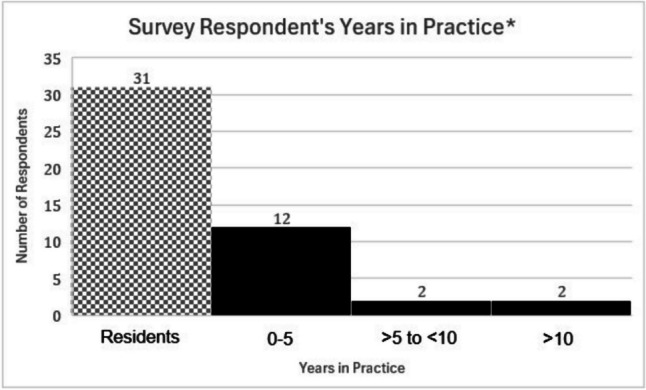
Distribution of years in practice for survey respondents. *13 respondents level of experience is unknown

The survey was started by 60 people (39.7% response rate) and 48 people completed the entire survey (31.8%). The respondents consisted of 31 residents (51.6%), 16 Faculty/APPs (26.7%), and 13 unknown (21.7%) who exited the survey prior to responding to the final question distinguishing residents from faculty/APPs. The distribution of survey respondents’ years of experience is outlined in Fig. 2.

**Fig. 3:**
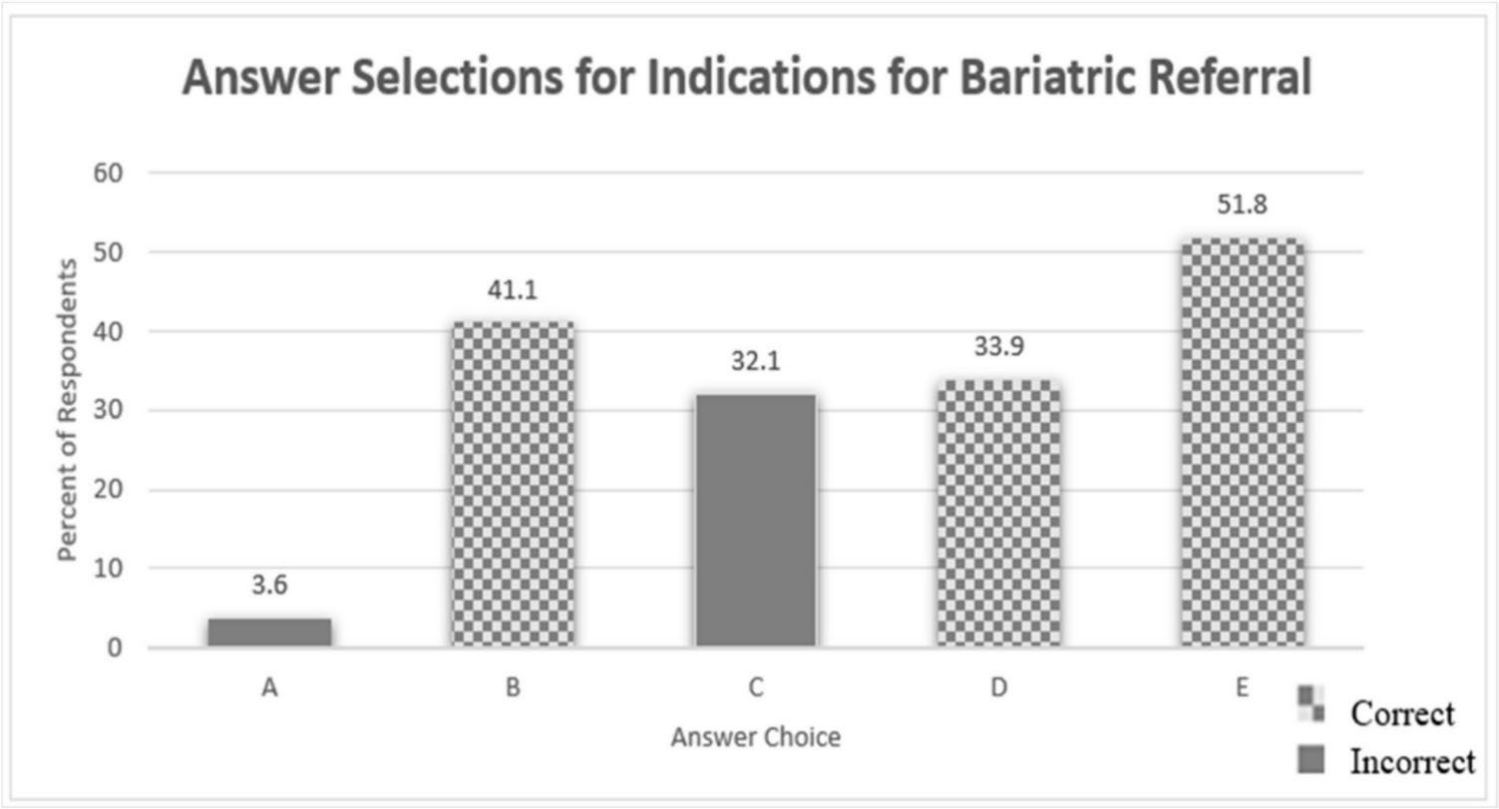
Percent of respondents selecting each answer choice for the following multiple-response question. Question 2: Based on current recommendations, which of the following is considered an indication for metabolic and bariatric surgery referral? Check all that apply: A, body mass index (BMI) ≥ 30 kg/m^2^, regardless of presence, absence, or severity of co-morbidities). B, BMI ≥ 35 kg/m^2^, regardless of presence, absence, or severity of co-morbidities). C, BMI ≥ 35 kg/m^2^, only in the presence of co-morbidities). D, BMI of 30–34.9 kg/m^2^ with associated type 2 diabetes). E, BMI of 30–34.9 kg/m.^2^ in patients who have not achieved substantial or durable weight loss or co-morbidity improvement using nonsurgical methods)

Ninety-five percent of the respondents indicated that they were not aware that the ASMBS and IFSO released updated guidelines in 2022. Question two was a multiple-response question asking respondents to identify all correct indications for bariatric surgery based on BMI. There were five response options, three of which were correct indications (Fig. 3). A total of 16.6% selected all three correct indications; 13.3% selected two of the three correct indications; 41.6% selected one of the three correct indications; 28.3% selected zero correct indications.

A total of 19.2% of respondents correctly answered a multiple-choice question that Asian patients have a lower BMI threshold required for referral based on the 2022 ASMBS and IFSO updated guidelines; 62% of respondents correctly answered that no medical trial of weight loss was required for patients with a BMI over 35 prior to bariatric referral; 66% of respondents correctly answered that a medical trial of weight loss was recommended for patients with Class 1 obesity (BMI of 30–34.9) prior to bariatric referral.

A total of 16.3% of respondents were able to correctly identify the expected excess weight loss following MBS on a multiple-choice question; 46.8% were able to correctly identify the percent of patients expected to achieve remission of diabetes following MBS.

A total of 31.9% of respondents indicated that they have referred a patient with Class 1 obesity (BMI 30–34.9) for MBS; 72.3% of respondents indicated that they do not feel like they have received adequate education on MBS indications and results during their training; 85.1% of respondents stated they would be interested in receiving additional education.

A subgroup analysis was performed comparing responses from those who stated they had referred a patient with class 1 obesity for bariatric surgery to those who had not. Those who had referred a patient for MBS (31.9% of respondents) performed similar to those who had not referred a patient for MBS, except for performing worse on the multiple-choice question asking if there is an ethnicity with a lower BMI threshold required for referral (*p* = 0.02). An additional subgroup analysis comparing resident responses to practicing faculty/APPs showed no significant difference in correct answers between residents and Faculty/APPs.

## Discussion

This study represents the first assessment of PCP MBS knowledge since the release of the 2022 updated ASMBS and IFSO guidelines. The main aim of this study was to assess if the 2022 update had been effectively distributed to PCPs. The most notable response in this regard was that 95% of respondents indicated that they were unaware that the 2022 update existed.

Many prior studies highlight a PCP knowledge deficit regarding MBS [6, 9, 10]. Conaty et al. found that lack of familiarity with bariatric procedures was the third most common barrier to referral for MBS in their survey-based study [6]. However, most prior studies asked PCPs to rate their comfort level with MBS knowledge and indications rather than asking them specific questions to demonstrate their knowledge. Our study asked specific questions regarding the indications and results of MBS to objectively identify the current PCP knowledge level. Some notable results on the questions regarding MBS indications included only 16.6% of respondents correctly identifying all three BMI-based MBS indications on a multiple-response question. On the same question, 28.3% of respondents were unable to select any correct indications.

Additionally, this study is novel for its inclusion of PCP residents to gain a more complete view of current PCP education. On subgroup analysis comparing resident responses to practicing PCPs, there was no significant difference in correct answers. There is minimal literature comparing attending to trainee knowledge. It is the authors’ opinion that this finding emphasizes that MBS as an effective treatment of obesity and obesity-related diseases has not been stressed at a trainee level, both in the past and present. It also further demonstrates poor dissemination of the updated ASMBS and IFSO guidelines to both PCPs and trainees.

While some of the low correct response rates can be attributed to the fact that 95% of respondents had not been aware of the 2022 updated guidelines prior to starting the survey, there were multiple questions regarding the expected results of MBS that were not based on the updated guidelines but had similar low correct response rates. Only 16.3% of respondents were able to correctly identify the expected excess weight loss following MBS on a multiple-choice question, and 46.8% of respondents were able to correctly identify the percent of patients expected to achieve remission of diabetes following MBS. These results emphasize the need for educational outreach to PCPs not only regarding the 2022 updated ASMBS and IFSO guidelines but also the current expected results following MBS.

On subgroup analysis, the 31.9% of respondents who have referred a patient with Class 1 obesity (BMI 30–34.9) for MBS performed no different on most questions and worse on one question when compared to those who had not referred a patient for MBS. This is an important finding because many MBS surgeons would assume that if a PCP is referring for MBS, they have a good understanding of the indications and expected outcomes, but our results indicate that this may not be the case. We suggest that this finding provides further motivation to establish regular communication between surgeons and PCPs, because if the surgeon was in the habit of updating referring PCPs with their recommendations and outcomes for their shared patients, it would provide an opportunity for continued education.

There are multiple limitations to this study to mention. This survey-based study is subject to some inherent response bias. The survey was started by 60 people (39.7% response rate), but only 48 people completed the entire survey (31.8%). This response rate is comparable and better in many cases, with similar MBS PCP survey-based studies. However, the small sample size at a single institution impacts its generalizability. While we believe that the inclusion of residents in the survey is novel and important for assessing the current state of PCP education, it can be considered a limitation that many of the respondents are trainees and not practicing PCPs. The questionnaire used in this study was not validated. The questions were designed using a literature review of similar survey studies as well as clinical knowledge; however, there is an unavoidable potential for bias whenever survey questions are written by the study investigators. In the interest of keeping the survey as short as possible to increase responses, specific demographics of the respondents such as age, gender, and BMI were not obtained which prevented more subgroup analysis of respondents. It should be noted that the purpose of the study was to better understand PCP and trainee knowledge within our own institution to better assess the need for educational outreach, which we believe was achieved.

Given that 95% of respondents had not heard of the 2022 updated guidelines, there is a clear opportunity to improve the distribution of these updated guidelines to our PCP colleagues. This study also indicates a gap in PCPs’ knowledge regarding the indications and results of MBS as illustrated above. Notably, 72.3% of respondents selected that they do not feel like they have received adequate education on MBS indications and results during their training, and 85.1% of respondents indicated that they would be interested in receiving additional education. Future directions for this project include providing education on MBS to our institutions’ family and internal medicine departments via lectures and grand rounds and surveying them following this education to track the education’s effectiveness. Of note, as mentioned in materials and methods, the survey itself provided the correct answers to each question with an explanation, thus introducing many of the most important points from the 2022 updated guidelines.

## Conclusion

We present the first assessment of PCP MBS knowledge since the release of the 2022 updated ASMBS and IFSO guidelines. This single-institution survey indicates a gap in PCPs’ knowledge regarding the updated guidelines and represents an opportunity for collaboration with our primary care colleagues to provide further education on MBS. Dissemination of guidelines is paramount to increasing access to MBS through PCP referral. By improving collaboration with PCPs, we can narrow the gap between those eligible and those who undergo MBS.

### Supplementary Information

Below is the link to the electronic supplementary material.

Supplementary file1 (PDF 55 KB)

## Data Availability

No datasets were generated or analysed during the current study.
