# Supplementary material for: Dissemination of the 2022 ASMBS and IFSO Guidelines for Bariatric Surgery: What Has Reached Primary Care Providers?
Source: Obes Surg. 2024 Aug 15;34(9):3252–7. doi: 10.1007/s11695-024-07449-1 (PMC11349814; doi:10.1007/s11695-024-07449-1)
Supplement: Supplementary file 1 — Supplementary file1 (PDF 55 KB) [file 11695_2024_7449_MOESM1_ESM.pdf]

Primary Care Physician Survey

Please complete the survey below.

Thank you!

This survey has 10 questions about updates to the indications for Bariatric and Metabolic Surgery and includes immediate explanations for each question. It takes about 5 minutes.

We appreciate you taking the Survey!

Question 1:

Were you aware that there was an update to the indications for Metabolic and Bariatric Surgery in 2022 by the American Society for Metabolic and Bariatric Surgery (ASMBS) and International Federation for the Surgery of Obesity and Metabolic Disorders (IFSO) ?

- ◯ Yes
- ◯ No

Question 2:

Based on current recommendations, which of the following is considered an indication for Metabolic and Bariatric surgery referral? Check all that apply:

- ☐ A: Body Mass Index (BMI) ≥30 kg/m2, regardless of presence, absence, or severity of co-morbidities
- ☐ B: BMI ≥35 kg/m2, regardless of presence, absence, or severity of co-morbidities
- ☐ C: BMI ≥35 kg/m2, only in the presence of co-morbidities
- ☐ D: BMI of 30-34.9 kg/m2 with associated type 2 Diabetes
- ☐ E: BMI of 30-34.9 kg/m2 in patients who have not achieved substantial or durable weight loss or co-morbidity improvement using nonsurgical methods

Question 2 Answer:

Answer: B, D, and E.

Explanation: Based on the 2022 American Society for Metabolic and Bariatric Surgery (ASMBS) and International Federation for the Surgery of Obesity and Metabolic Disorders (IFSO) update, metabolic and bariatric surgery (MBS) is recommended for:

- Individuals with a BMI ≥35 kg/m2, regardless of presence, absence, or severity of co-morbidities
- Patients with BMI of 30-34.9 kg/m2 with associated type 2 Diabetes
- MBS should be considered for individuals with metabolic disease and BMI of 30-34.9 kg/m2 who do not achieve substantial or durable weight loss or co-morbidity improvement using nonsurgical methods.

These are new updates from the prior 1991 guidelines where referral was recommended for BMI ≥35 kg/m2 with comorbidities or BMI ≥40 kg/m2 with or without comorbidities (1).

Question 3:

Based on current recommendations, are there any ethnicities for which the BMI thresholds for referral for metabolic and bariatric surgery are lower?

- ◯ A: Yes, African American
- ◯ B: Yes, Asian
- ◯ C: Yes, Native Americans
- ◯ D: No, the BMI thresholds for referral are the same for all ethnicities

Question 3 Answer:

Answer: B

Explanation: Based on the 2022 American Society for Metabolic and Bariatric Surgery (ASMBS) and International Federation for the Surgery of Obesity and Metabolic Disorders (IFSO) update, BMI thresholds should be adjusted in the Asian population such that:

- BMI ≥25 kg/m2 suggests clinical obesity
- BMI ≥27.5 kg/m2 should be offered MBS

This adjustment is based on evidence that in the Asian population the prevalence of diabetes and cardiovascular disease is higher at a lower BMI than in the non-Asian populations (1).

Question 4:

Based on current recommendations, is it necessary for patients with (BMI) ≥35 kg/m2 to undergo a trial of medical weight loss prior to bariatric referral?

◯ Yes
◯ No

Question 4 Answer:

Answer: No

Explanation: It has been demonstrated that current nonsurgical treatment options for patients with BMI ≥35 kg/m2 are ineffective in achieving a substantial and sustained weight reduction. Given the abundance of data demonstrating safety, efficacy, and cost-effectiveness of MBS in patients with BMI ≥35 kg/m2, MBS should be strongly recommended in these patients regardless of co-morbidities or prior weight loss attempts (1).

Question 5:

Based on current recommendations, should patients with BMI of 30-34.9 kg/m2 with associated metabolic disease undergo a trial of medical weight loss prior to bariatric referral?

◯ Yes
◯ No

Question 5 Answer:

Answer: Yes

Explanation: Medical weight loss is considered to have greater durability in individuals with BMI < 35 kg/m2 than individuals with BMI ≥35 kg/m2, and thus it is recommended that a trial of nonsurgical therapy is attempted before considering surgical treatment. However, if attempts at treating obesity and obesity-related co-morbidities have not been effective, MBS should be considered (1).

Question 6:

Based on current data, what is an approximate amount of weight loss to be expected following Metabolic and Bariatric Surgery?

- ◯ A: 30% of excess weight
- ◯ B: 40% of excess weight
- ◯ C: 50% of excess weight
- ◯ D: 60% of excess weight
- ◯ E: 70% of excess weight

Question 6 Answer:

Answer: D

Explanation:
- Overall weight loss with MBS is consistently reported at greater than 60% percent excess weight loss, with some variation depending on the specific operation performed.
- The durability of weight loss at 5, 10, and 20 years after surgery has been consistently demonstrated in multiple studies
- MBS is proven superior to diet, exercise, and other lifestyle interventions in attaining significant and durable weight loss (1).

Question 7:

Based on current data, what is the expected diabetes remission rate (Hemoglobin A1c < 6.5%) following a bariatric surgery on patients with BMI of 30-34.9 kg/m2?

- ◯ A: 25% of patients expected to achieve diabetes remission
- ◯ B: 35% of patients expected to achieve diabetes remission
- ◯ C: 45% of patients expected to achieve diabetes remission
- ◯ D: 55% of patients expected to achieve diabetes remission

Question 7 Answer:

Answer: D

Explanation:
In a large systematic review examining the effect of a variety of metabolic and bariatric surgeries on patients with type 2 diabetes and class 1 obesity (BMI of 30-34.9 kg/m2), the average rate of diabetes remission (HbA1C < 6.5% without medications) was 55% (95% confidence interval, 44%–65%) at 12 months. This varied based on the procedure performed:
- Adjustable gastric band, 33% (rarely performed today)
- Single-anastomosis gastric bypass, 49%
- Sleeve gastrectomy, 54%
- Roux-en-Y-Gastric Bypass, 64%
- Biliopancreatic Diversion, 70%

This along with multiple other high quality studies demonstrating durable improvement in diabetes control following MBS resulted in the new recommendation for MBS in patients with BMI of 30-34.9 kg/m2 with associated type 2 Diabetes (2,3).

Question 8:

Have you personally referred a patient for consideration of metabolic or bariatric surgery with class 1 obesity (BMI of 30-34.9 kg/m2)?

◯ Yes
◯ No

Question 9:

Do you feel like you received adequate education on Bariatric surgery indications and results during your training?

◯ Yes
◯ No

Question 10:

Would you be interested in receiving additional education on Bariatric surgery indications and results?

◯ Yes
◯ No

Final Question:

How many years have you been a primary care provider? Please enter a number (if you are a resident please enter "0"). __________________________________

Works Cited

Eisenberg D, Shikora SA, Aarts E, Aminian A, Angrisani L, Cohen RV, De Luca M, Faria SL, Goodpaster KPS, Haddad A, Himpens JM, Kow L, Kurian M, Loi K, Mahawar K, Nimeri A, O'Kane M, Papasavas PK, Ponce J, Pratt JSA, Rogers AM, Steele KE, Suter M, Kothari SN. 2022 American Society for Metabolic and Bariatric Surgery (ASMBS) and International Federation for the Surgery of Obesity and Metabolic Disorders (IFSO): Indications for Metabolic and Bariatric Surgery. Surg Obes Relat Dis. 2022 Dec;18(12):1345-1356. doi: 10.1016/j.soard.2022.08.013. Epub 2022 Oct 21. PMID: 36280539.

Parikh M, Issa R, Vieira D, McMacken M, Saunders JK, Ude-Welcome A, Schubart U, Ogedegbe G, Pachter HL. Role of bariatric surgery as treatment for type 2 diabetes in patients who do not meet current NIH criteria: a systematic review and meta-analysis. J Am Coll Surg. 2013 Sep;217(3):527-32. doi: 10.1016/j.jamcollsurg.2013.04.023. Epub 2013 Jul 24. PMID: 23890843.

Aminian A, Chang J, Brethauer SA, Kim JJ; American Society for Metabolic and Bariatric Surgery Clinical Issues Committee. ASMBS updated position statement on bariatric surgery in class I obesity (BMI 30-35 kg/m2). Surg Obes Relat Dis. 2018 Aug;14(8):1071-1087. doi: 10.1016/j.soard.2018.05.025. Epub 2018 Jun 9. PMID: 30061070.
